# Influence of Tattoo Ink on Hepatitis C Virus Infectiousness

**DOI:** 10.1093/ofid/ofz047

**Published:** 2019-02-13

**Authors:** Patrick Behrendt, Janina Brüning, Daniel Todt, Eike Steinmann

**Affiliations:** 1 Institute of Experimental Virology, Twincore, Centre for Experimental and Clinical Infection Research; a joint venture between the Medical School Hannover (MHH) and the Helmholtz Centre for Infection Research (HZI) Hannover, Germany; 2 Department of Gastroenterology, Hepatology and Endocrinology, Hannover Medical School, Hannover, Germany; 3 German Center for Infection Research, partner site Hannover-Braunschweig, Hannover, Germany; 4 Department for Molecular and Medical Virology, Medical Faculty, Ruhr University Bochum, Bochum, Germany

**Keywords:** hepatitis C virus, transmission, prevention, viral stability

## Abstract

Hepatitis C virus (HCV) is a blood-borne virus and is most frequently transmitted through large or repeated direct percutaneous exposures to infected blood. The 2 most common exposures associated with transmission of HCV are blood transfusion and intravenous drug abuse. The association between HCV transmission and other suspected risk factors such as tattooing is more controversial. Although HCV can survive for days to weeks in suspension or on inanimate surfaces, its stability in tattooing supplies remains elusive. Here, we analyzed the influence of tattoo ink on HCV infectiousness.

Hepatitis C virus (HCV) belongs to the hepacivirus genus within the *Flaviviridae* family. HCV infections become chronic in up to 80% of the cases, resulting in approximately 71 million people worldwide suffering from chronic hepatitis C [1]. The virus causes a progressive viral hepatitis leading to liver steatosis, fibrosis, cirrhosis, or hepatocellular carcinoma and ultimately to liver failure. Until 2014, anti-HCV treatment was mainly limited to interferon-based therapies, with cure rates between 30% and 80% [2]. However, the development of interferon-free regimens incorporating direct-acting antivirals (DAAs) targeting the HCV nonstructural proteins led to cure rates exceeding 95% in real-life settings. However, once a patient has cured an HCV infection, this does not protect from re-infection.

HCV is a blood-borne virus, which is mainly transmitted parenterally due to the use of unsafe injection material. Currently, injection drug use (IDU) is the leading cause of transmission, accounting for 60% of new cases each year [3]. However, around 20% of incident cases have no history of IDU or other parental exposures. In contrast to the strong evidence implicating IDU in HCV acquisition, the association between HCV transmission and other suspected risk factors such as tattooing is more controversial [4]. Although some studies have demonstrated an association between tattoos and HCV infection, others have not [5]. Tattooing requires injection of ink pigments into the dermal layer of the skin by puncturing the skin hundreds of times a second. As tattoo equipment comes into contact with blood and body fluids, HCV may be transmitted whenever the instruments are used several times without being sterilized or without proper hygiene measures. Additionally, transmission of HCV could occur at distinct stages of tattooing, for example, during reuse of needles or reuse of ink that is contaminated with HCV-positive blood. As tattoo dyes are not kept in sterile containers, they could be carriers for transmission of HCV [6]. No studies or data are available on the survival of HCV in tattooing utensils. Therefore, we analyzed the risk of HCV transmission via contaminated tattoo ink.

## METHODS

HCV chimeric Jc1 virus was generated in the human hepatoma cell line Huh-7.5, as previously described [7].

Huh-7.5 cells were cultured in Dulbecco’s modified Eagle medium (Invitrogen) with 10% fetal bovine serum, 1× nonessential amino acids (Invitrogen), 100 µg/mL streptomycin (Invitrogen), and 100 IU/mL penicillin (Invitrogen).

For the suspension test, 9 parts by volume of the test virus suspension were mixed with 1 part by volume of different tattoo inks. Titers of infectious virus were determined using a limiting dilution assay on Huh-7.5 cells, and tissue culture infectious dose 50 (TCID50) was determined as described elsewhere [8].

Stainless steel discs for carrier assay were prepared as previously described [9]. One part of the respective tattoo ink was mixed with 1 part of the virus suspension, pipetted in the center of each pretreated carrier, and dried under a laminar flow for about 1–3 hours at room temperature. The virus was recovered as previously described [9], and TCID50 was determined as mentioned above.

## RESULTS

**Figure 1.**
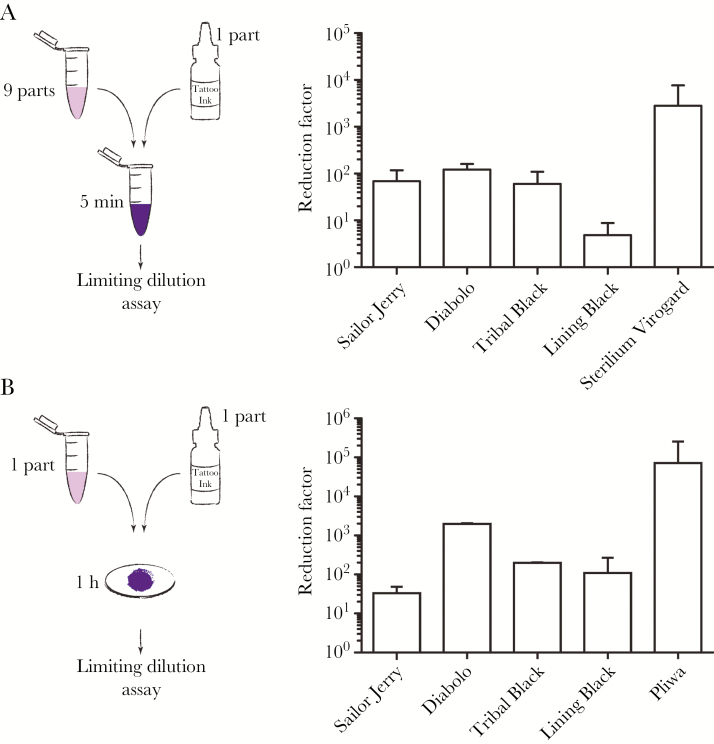
Virucidal activity of tattoo ink against hepatitis C virus (HCV). A, Four different tattoo inks (Sailor Jerry, Diabolo, Tribal Black, Lining Black) and 1 commercial hand disinfectant (Sterilium virugard) were tested in a quantitative suspensions assay for the efficiency to inactivate HCV. Experiments were carried out by mixing 1 part of test virus suspension with 9 parts of the different tattoo inks or the hand antiseptic. After incubation for 5 minutes, test mixtures were immediately serially diluted and titrated in microplates. Viral titers were determined by a limiting dilution assay. Titer reduction, calculated as the difference between the virus titer of the Phosphate-buffered saline (PBS) control and the respective ink, is presented as reduction factor (RF). B, Four different tattoo inks and 1 commercial surface disinfectant (Pliwa) were tested in a carrier assay. A mixture of 1 part virus and 1 part ink was dried under a laminar flow for 1 hour. After drying, discs were transferred into 25-mL plastic vial holders, 1 mL of cell culture medium was added, and viral infectivity recovered from the disk was determined. Infectivity is expressed relative to the eluted HCV infectious particles when virus was dried in the presence of PBS. Mean values and standard deviations of 3 independent experiments are shown.

To estimate the risk of HCV transmission via contaminated tattoo ink, we incubated cell culture–derived infectious HCV with 4 different commercially available tattoo inks (Sailor Jerry, Diabolo, Tribal Black, Lining Black) and a reference hand disinfectant (Sterilium virugard) for 5 minutes in a suspension assay. As depicted in Figure 1A, different tattoo inks did not stimulate, but rather reduced HCV infectiousness by 2 orders of magnitude, although 1 brand exerted a lower but still detectable inactivation effect (Figure 1A). Next, we tested the degree of cross-contamination of inanimate surfaces (eg, needles) from contaminated tattoo ink as a measure of the risk for virus cross-transmission by tattooing procedures. To this end, we dried a mixture of HCV and tattoo inks on small steel discs and determined viral infectivity adherent to these disks as described previously [9]. As expected, a commercial disinfectant (Pliwa) displayed the strongest virucidal effect. Moreover, the tattoo inks reduced HCV titers by 50- to 1000-fold depending on the brand (Figure 1B).

## DISCUSSION

To date, there is no definitive evidence that HCV infection occurs through tattooing when sterile material is used. Although no outbreaks of HCV infection have been observed that originate from professional tattoo parlors, case reports of acute HCV infection from tattooing in prison suggest that tattooing could be a mode of transmission [10, 11]. The data obtained in our study indicate that components of tattoo ink partially inactivate HCV and that different ink brands substantially differ in their virucidal activity. Thus, unlike some anesthetic agents, an increase of HCV stability in the presence of tattoo ink could not be observed and therefore does not facilitate infection [12, 13]. However, because of the increasing prevalence of tattooing, particularly among youths, awareness campaigns should still highlight the danger of transmitting blood-borne infections such as HCV, regardless of the venue of placement.

In summary, incubation of HCV in tattoo ink reduces HCV infectiousness, while residual virus is still detectable, indicating that HCV transmission is not facilitated through virus–ink mixtures. Notably, ink brands differ in virucidal activity. Thus, selection of ink brands with high antiviral activity may reduce the risk of HCV transmission via this route.

## References

[1] WHO. Global hepatitis report. 2017 https://apps.who.int/iris/bitstream/handle/10665/277005/WHO-CDS-HIV-18.46-eng.pdf?ua=1. Accessed September 30, 2018.

[2] Heim MH Innate immunity and HCV. J Hepatol 2013; 58:564–74. 23063572 10.1016/j.jhep.2012.10.005

[3] Alter MJ, Kruszon-Moran D, Nainan OV, et al The prevalence of hepatitis C virus infection in the United States, 1988 through 1994. N Engl J Med 1999; 341:556–62. 10451460 10.1056/NEJM199908193410802

[4] Carney K, Dhalla S, Aytaman A, et al. Association of tattooing and hepatitis C virus infection: a multicenter case-control study. Hepatology 2013; 57:2117–23. 23315899 10.1002/hep.26245

[5] Tohme RA, Holmberg SD Transmission of hepatitis C virus infection through tattooing and piercing: a critical review. Clin Infect Dis 2012; 54:1167–78. 22291098 10.1093/cid/cir991 PMC4613802

[6] Jafari S, Copes R, Baharlou S, et al. Tattooing and the risk of transmission of hepatitis C: a systematic review and meta-analysis. Int J Infect Dis 2010; 14:e928–40. 20678951 10.1016/j.ijid.2010.03.019

[7] Pietschmann T, Kaul A, Koutsoudakis G, et al. Construction and characterization of infectious intragenotypic and intergenotypic hepatitis C virus chimeras. Proc Natl Acad Sci U S A 2006; 103:7408–13. 16651538 10.1073/pnas.0504877103 PMC1455439

[8] Lindenbach BD, Evans MJ, Syder AJ, et al. Complete replication of hepatitis C virus in cell culture. Science 2005; 309:623–6. 15947137 10.1126/science.1114016

[9] Doerrbecker J, Friesland M, Ciesek S, et al. Inactivation and survival of hepatitis C virus on inanimate surfaces. J Infect Dis 2011; 204:1830–8. 22013220 10.1093/infdis/jir535 PMC3247810

[10] Post JJ, Dolan KA, Whybin LR, et al. Acute hepatitis C virus infection in an Australian prison inmate: tattooing as a possible transmission route. Med J Aust 2001; 174:183–4. 11270759 10.5694/j.1326-5377.2001.tb143214.x

[11] Tsang TH, Horowitz E, Vugia DJ Transmission of hepatitis C through tattooing in a United States prison. Am J Gastroenterol 2001; 96:1304–5. 11316197 10.1111/j.1572-0241.2001.03728.x

[12] Behrendt P, Doerrbecker J, Riebesehl N, et al. Stability and transmission of hepatitis C virus in different anesthetic agents. Am J Infect Control 2013; 41: 942–3. 23523523 10.1016/j.ajic.2013.01.016

[13] Steinmann E, Ciesek S, Friesland M, et al. Prolonged survival of hepatitis C virus in the anesthetic propofol. Clin Infect Dis 2011; 53:963–4. 21880582 10.1093/cid/cir530

